# An exonic splicing enhancer mutation in *DUOX2* causes aberrant alternative splicing and severe congenital hypothyroidism in Bama pigs

**DOI:** 10.1242/dmm.036616

**Published:** 2019-01-15

**Authors:** Chunwei Cao, Ying Zhang, Qitao Jia, Xiao Wang, Qiantao Zheng, Hongyong Zhang, Ruigao Song, Yongshun Li, Ailing Luo, Qianlong Hong, Guosong Qin, Jing Yao, Nan Zhang, Yanfang Wang, Hongmei Wang, Qi Zhou, Jianguo Zhao

**Affiliations:** 1 State Key Laboratory of Stem Cell and Reproductive Biology, Institute of Zoology, Chinese Academy of Sciences, Chaoyang District, Beijing 100101, China; 2 Savaid Medical School, University of Chinese Academy of Sciences, Beijing 100049, China; 3 College of Life Science, Qufu Normal University, Qufu 273165, China; 4 Institute of Animal Science, Chinese Academy of Agricultural Sciences, Beijing 100193, China

**Keywords:** Pigs, Animal model, ENU, Exome sequencing, Congenital hypothyroidism

## Abstract

Pigs share many similarities with humans in terms of anatomy, physiology and genetics, and have long been recognized as important experimental animals in biomedical research. Using an N-ethyl-N-nitrosourea (ENU) mutagenesis screen, we previously identified a large number of pig mutants, which could be further established as human disease models. However, the identification of causative mutations in large animals with great heterogeneity remains a challenging endeavor. Here, we select one pig mutant, showing congenital nude skin and thyroid deficiency in a recessive inheritance pattern. We were able to efficiently map the causative mutation using family-based genome-wide association studies combined with whole-exome sequencing and a small sample size. A loss-of-function variant (c.1226 A>G) that resulted in a highly conserved amino acid substitution (D409G) was identified in the *DUOX2* gene. This mutation, located within an exonic splicing enhancer motif, caused aberrant splicing of *DUOX2* transcripts and resulted in lower H_2_O_2_ production, which might cause a severe defect in thyroid hormone production. Our findings suggest that exome sequencing is an efficient way to map causative mutations and that *DUOX2*^D409G/D409G^ mutant pigs could be a potential large animal model for human congenital hypothyroidism.

## Abstract (precis)

**Summary:** Here, we show that an exonic splicing enhancer variant in *DUOX2* (c.1226 A>G) causes aberrant splicing of *DUOX2* transcripts, resulting in lower H_2_O_2_ production, to cause severe congenital hypothyroidism in Bama pigs.

## INTRODUCTION

Pigs are considered to be one of the major livestock and are increasingly used in biomedical research (Swindle et al., 2012). Pig models possess many advantages as human disease models because they share many anatomical, morphological and physiological similarities with humans (Nunoya et al., 2007). Thus far, many pig models for human diseases – such as Alzheimer's disease, retinitis pigmentosa, spinal muscular atrophy, cardiovascular diseases and cancers – have been created for the purpose of biomedical research (Prather et al., 2013). Furthermore, the pigs are regarded as ideal organ donors for xenotransplantation into humans (Lai et al., 2002), and as high efficiency bioreactors for the production of pharmaceuticals (Zhao et al., 2015). It is likely that, with the rapid development of genome engineering tools, pigs will become an increasingly important experimental animal for biomedical research.

N-ethyl-N-nitrosourea (ENU) mutagenesis is a powerful forward-genetic approach for discovering gene function and generating animal models for human disorders (Acevedo-Arozena et al., 2008). Compared with gene-driven or reverse-genetic approaches, chemical mutagenesis has many advantages. ENU-induced mutagenesis does not require any prior knowledge or assumptions about the genetic basis of the genes involved. Unlike conventional gene knockout approaches, which result in null alleles, ENU mutagenesis primarily induces point mutations, which are similar to those that arise naturally (Oliver and Davies, 2012). To date, ENU mutagenesis screening has been widely performed to identify animal models for the study of gene functions and human diseases in *Caenorhabditis* *elegans* (De Stasio and Dorman, 2001), *Drosophila* (Choi et al., 2009), zebrafish (Wienholds et al., 2003) and mice (Hrabe de Angelis et al., 2000). We recently reported, for the first time, a large-scale ENU mutagenesis in Chinese Bama pigs, and demonstrated the effectiveness of ENU mutagenesis in a large mammalian species (Hai et al., 2017). Through systemic phenotyping screening, an abundance of mutants exhibiting a broad range of phenotypes were identified in our mutagenesis program. These pig mutants were first confirmed to inherit stably in either a dominant or a recessive pattern, then genetics and genomics analysis were performed to map the causative genes that were responsible for the mutant traits. However, causal mutation mapping using genetic crosses has traditionally been considered a complex and multistep procedure (Schneeberger, 2014), and it remains quite challenging to efficiently isolate the causative mutations in our mutant pedigrees. The challenge is possibly a result of the heterogeneity of the genetic background (a large number of ENU-induced mutations introduced into the genome), the relatively smaller sample size and the low density of single-nucleotide polymorphism (SNP) markers in commercial genotyping chips (Ramos et al., 2009; Ai et al., 2013). Notably, the wide application of next-generation, high-throughput sequencing approaches, such as whole-genome and whole-exome sequencing, has dramatically increased the efficiency of causative gene identification, even in complex genetic backgrounds (Schneeberger, 2014; Jamuar and Tan, 2015; Boycott et al., 2013). Using these high-throughput sequencing methods, the gene discovery process has become much more straightforward in human and mice (Fairfield et al., 2011; Goh and Choi, 2012). However, the feasibility and effectiveness of whole-exome sequencing for the identification of causative mutations in ENU-mutagenized pigs has not been estimated previously.

In this study, we focus on a pig mutant line generated by ENU mutagenesis and aim to investigate the genetic basis of the mutant phenotype of congenital hypothyroidism. Our study confirms that whole-exome sequencing combined with family-based whole-genome association studies (GWAS) is a cost-efficient way to identify causative mutations in the ENU mutant pedigree. Furthermore, the identified causal mutation, c.1226 A>G, in *DUOX2* is located in an exonic splicing enhancer (ESE) motif and causes aberrant splicing of the *DUOX2* transcripts, dubbed *DUOX2a* and *DUOX2b*. The newly generated shorter isoform, *DUOX2b*, may play a role in severe thyroid hormone deficiency in pigs. Our findings indicate that the *DUOX2*^D409G/D409G^ mutant pigs could be used as a potential large animal model for human congenital hypothyroidism.

## RESULTS

### Identification of an ENU-induced pig mutant line with an autosomal recessive inheritance pattern

**Fig. 1.**
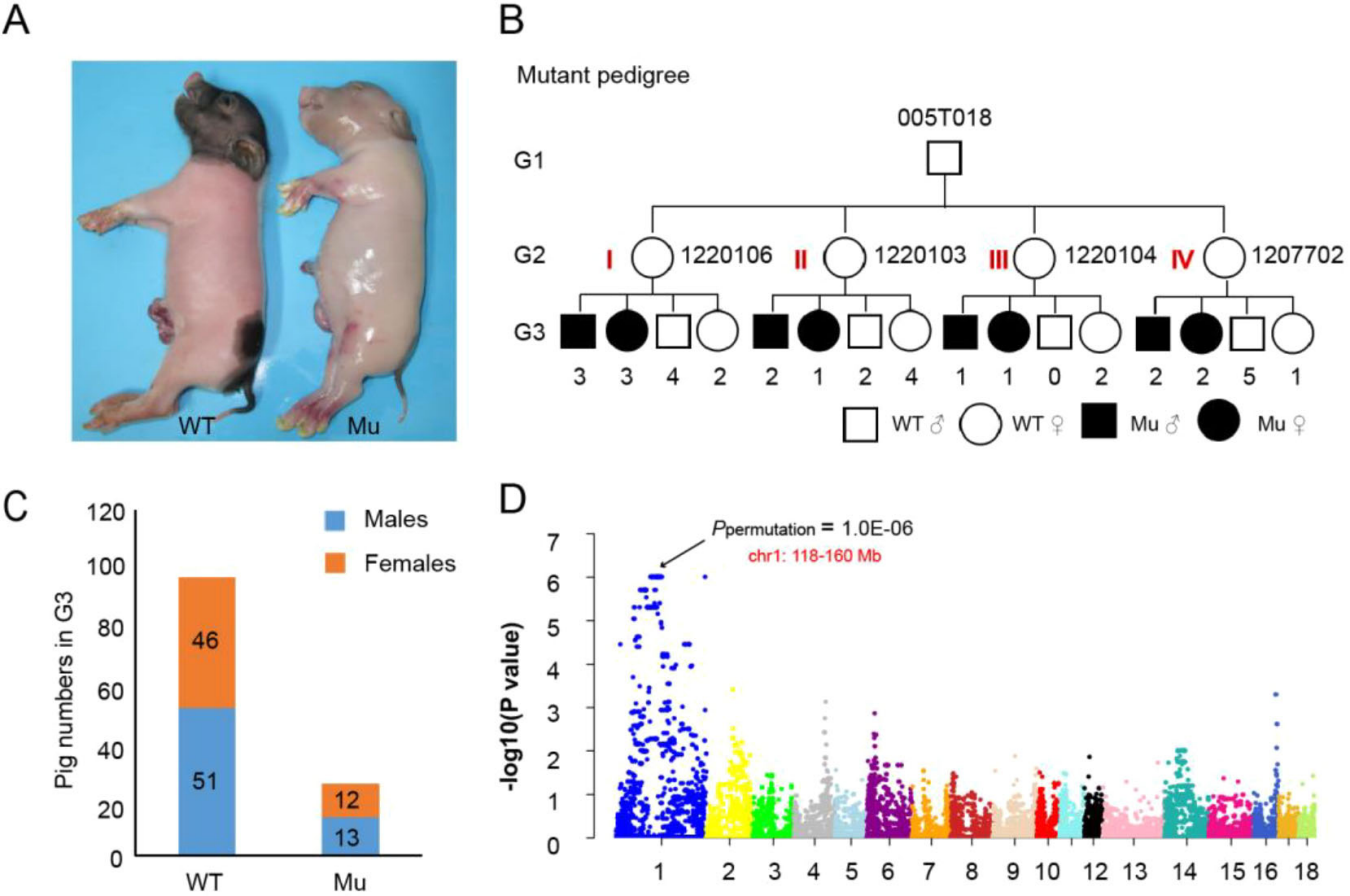
**Family-based GWAS of the mutant pedigree revealed a unique significant signal.** (A) The mutant created by ENU mutagenesis [Mu, shown next to wild type (WT)]. (B) The simplified three-generation pedigree chart. Four independent families were selected in the mutant pedigree, and the numbers below the squares and circles represent the number of pigs corresponding to each group in G3. (C) The distribution of mutant and wild-type pigs in the G3 population of the whole pedigree. (D) Family-based GWAS revealed a unique significant signal on chromosome 1: 118-160 Mb region.

We previously performed a large-scale ENU mutagenesis screen in Chinese Bama miniature pigs, and one mutant pig displaying congenital nude skin was identified (Fig. 1A). Further analysis of the mutant pedigree confirmed that this mutant trait inherits in an autosomal recessive pattern (Fig. 1B,C). To identify the causative gene responsible for the mutant phenotype, family-based GWAS were performed in 15 mutant pigs and 25 wild-type pigs from four selected families of the mutant pedigree (Fig. 1B; Table S1). The results indicated that, among the whole genome, only one genetic locus on chromosome 1 (118-160 Mb) showed genome-wide statistical significance (Fig. 1D; Table S2), which included 374 annotated genes from the Genome Data Viewer (GDV).

### Identifying ENU-induced lesions in the *DUOX2* gene

**Fig. 2.**
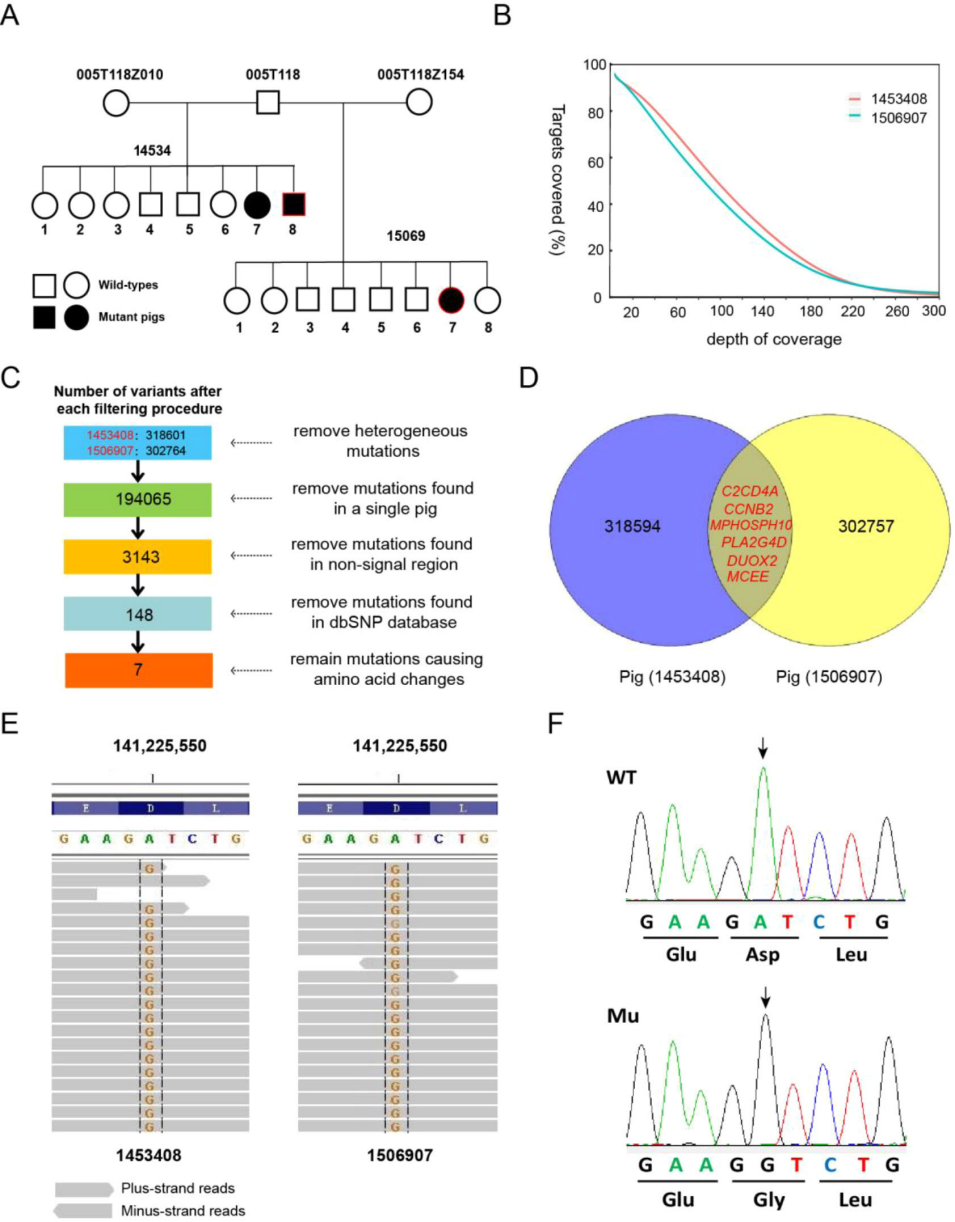
**Identification of the causal mutation using whole-exome sequencing.** (A) The mutant trait was inherited in a recessive pattern, and two mutant pigs (ID: 1453408 and 1506907, marked in red) were subjected to whole-exome sequencing analysis. (B) Coverage of sequence reads over the exome targets in two pigs. The results showed that more than 90% of the target region was covered by more than 20 reads. (C) A stepwise mutation filtering procedure was established to isolate the causative mutation. (D) Six candidate genes meeting the screening criteria were detected by exome sequencing. (E) A missense mutation in the *DUOX2* gene (p.D409G) was identified by whole-exome sequencing, which co-segregated with the mutant phenotype in the whole pedigree. (F) Validation of the *DUOX2* p.D409G mutation using Sanger sequencing.

To filter out causative mutations from genomic intervals and efficiently eliminate unrelated variants, two independent mutant pigs (ID: 1453408 and 1506907) were selected for whole-exome sequencing (Fig. 2A). Throughout the whole exome, the read depth statistics showed that more than 90% of target sequences are covered with a minimum depth of 20×, indicating that the target sequences are well covered in our sequencing analysis (Fig. 2B). Following a designed variant detection pipeline and a stepwise filtering procedure, the sequencing and bioinformatics analysis (Fig. 2C,D) ultimately revealed seven non-synonymous mutations in six candidate genes that met the entire filtering criteria (Table 1). Segregation analysis of these mutations indicated that only the mutation in the *DUOX2* gene (c.1226 A>G), but not other variants, completely co-segregated with the mutant phenotype in the family [all mutants were homozygous for the mutant alleles (GG), whereas other pigs exhibiting the normal phenotype were AA or AG genotypes] (Fig. 2E,F; Table S3). Moreover, we found that this mutation was not observed in other laboratory pedigrees or in commercial pig breeds (Table S3), implying that the mutation was specifically produced by ENU mutagenesis. Together, these results suggest that the *DUOX2* c.1226 A>G mutation might be the causative mutation for this mutant phenotype.

**Table 1.**
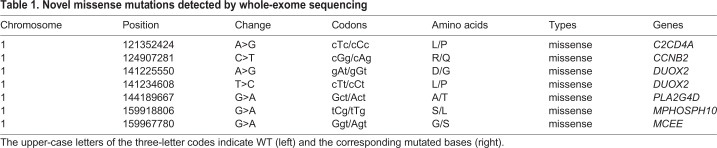
**Novel missense mutations detected by whole-exome sequencing**

### Homozygous loss of function of *DUOX2* (p.D409G) leads to thyroid hormone deficiency in pigs

**Fig. 3.**
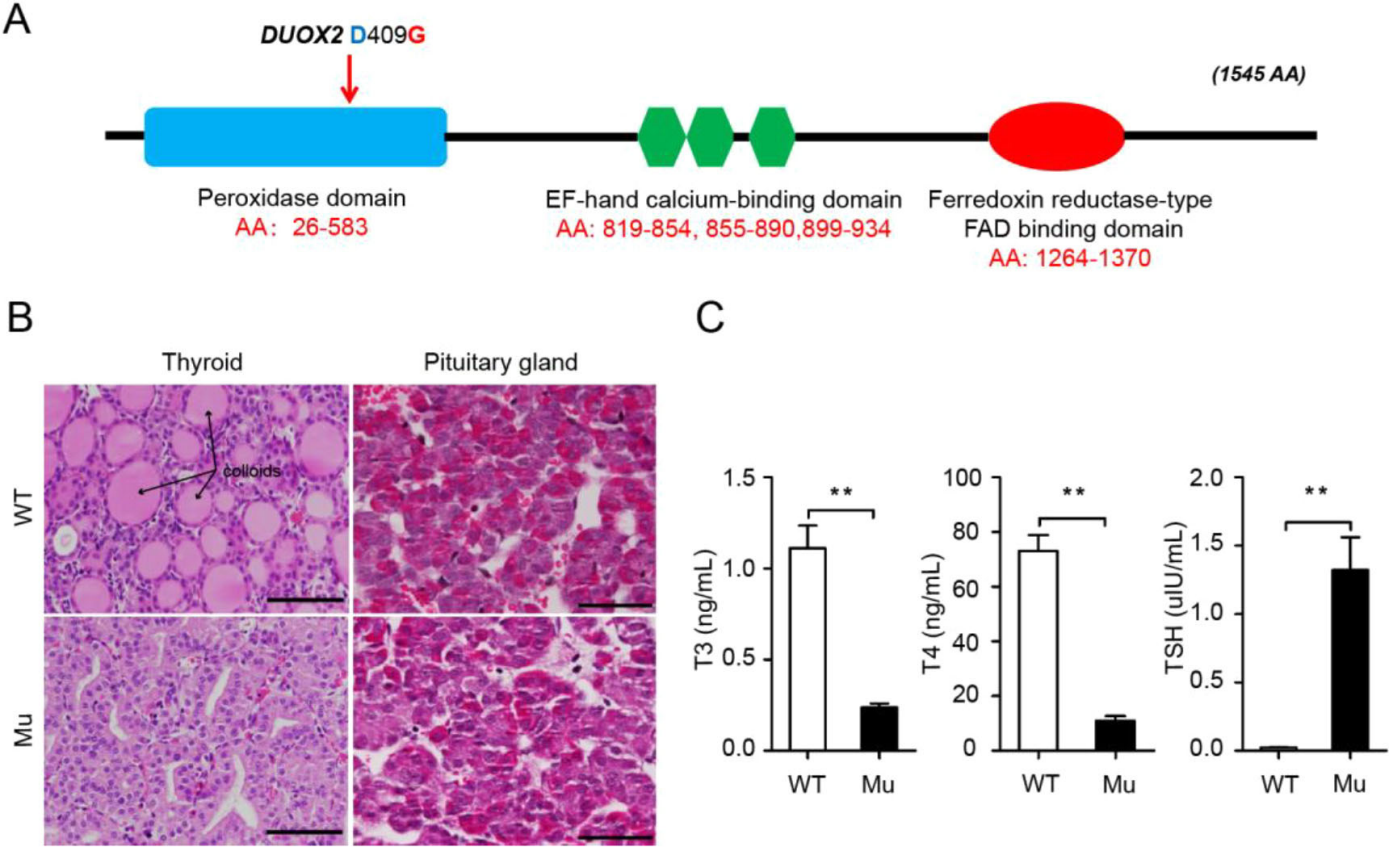
**The mutants exhibit congenital hypothyroidism.** (A) The mutation was located in the peroxidase-like domain. (B) Histological section analysis indicated abnormalities in the thyroid and pituitary glands, which are associated with thyroid hormone production. Scale bars: 50 μm. (C) Measurement of thyroid hormones in the peripheral blood showed that the thyroid hormones of T3 and T4 were significantly decreased in mutant pigs, whereas TSH was markedly elevated (mean±s.d.; Student's *t*-test; **P<0.01; *n*=6 in each group). AA, amino acids.

*In silico* analysis of DUOX2 protein sequences indicated that the mutated residue was located in the peroxidase-like domain of DUOX2 (Fig. 3A). Furthermore, the p.D409G mutation was predicted to have a deleterious or damaging effect on protein function by SIFT and PolyPhen servers. As *DUOX2* mutations have been reported to play a pathogenic role in human congenital hypothyroidism (Moreno et al., 2002), we thus investigated whether p.D409G also causes thyroid hormone deficiency in pigs. As indicated by the histological section of the thyroid gland (Fig. 3B), we observed almost complete disappearance of colloids (a proteinaceous depot of thyroid hormone precursor) in the mutant pigs. Simultaneously, the pathological analysis of the anterior pituitaries showed dysplasia, with many abnormal cells in mutant pigs but not their wild-type (WT) littermates (Fig. 3B). These data suggest that abnormal thyroid hormone synthesis might occur in the mutant pigs. Accordingly, the released thyroid hormones triiodothyronine (T3) and thyroxine (T4) were significantly decreased in the mutant pigs, whereas thyroid-stimulating hormone (TSH) was compensatorily elevated (Fig. 3C), suggesting that severe thyroid hormone deficiency occurred.

### The p.D409G mutation in *DUOX2* impairs the production of H_2_O_2_

**Fig. 4.**
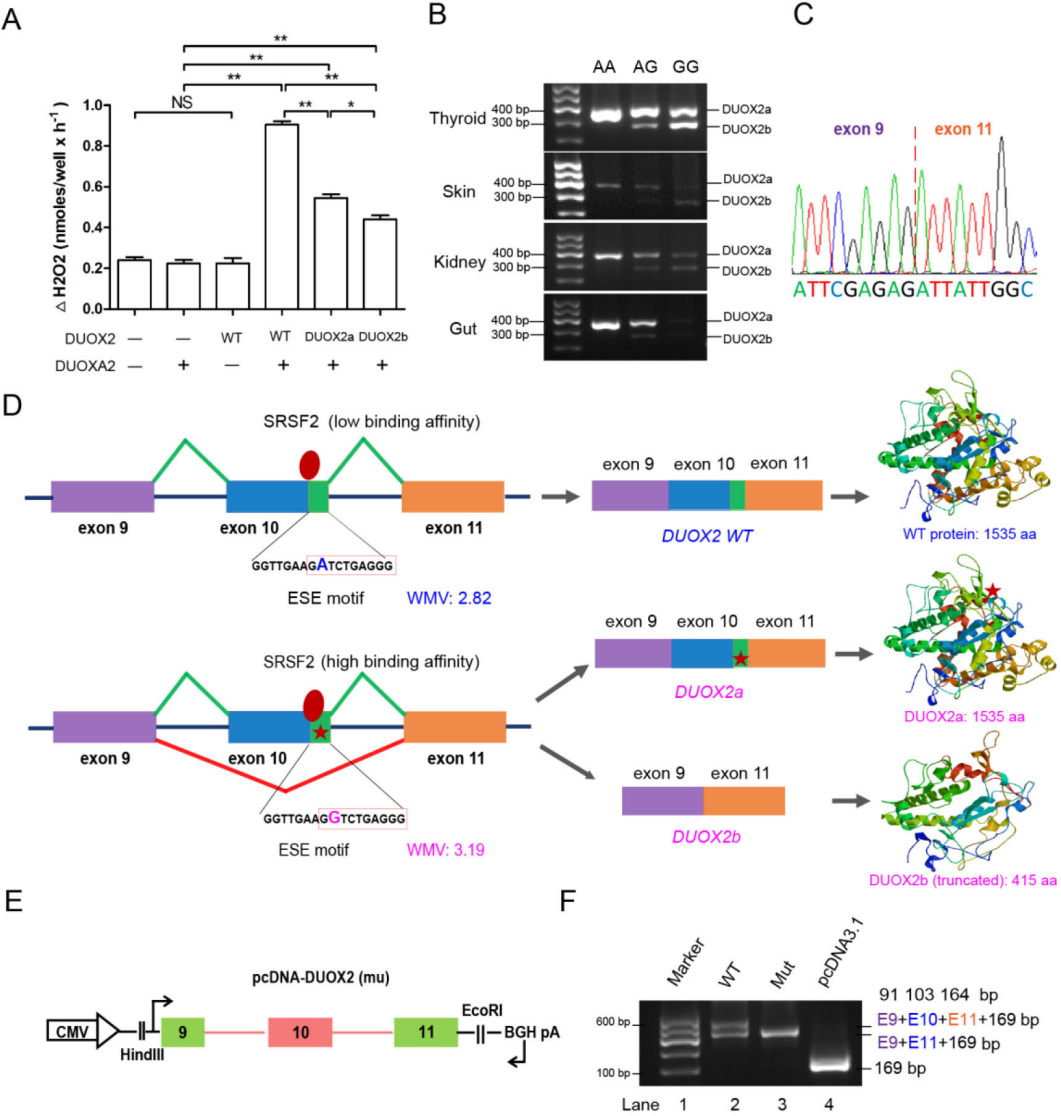
**The p.D409G mutation causes aberrant splicing of *DUOX2* transcripts.** (A) The H_2_O_2_ analysis showed that both mutant *DUOX2a* and *DUOX2b* transcripts showed significantly decreased H_2_O_2_ production compared with the WT transcript. The DUOX2b group produced a significantly lower level of H_2_O_2_ than that produced by the DUOX2a group (mean±s.d.; Student's *t*-test; NS, non-significant; *P<0.05, **P<0.01; *n*=6 in each group). (B) The RT-PCR products of multiple tissues (thyroid, skin, kidney and gut) were analyzed by agarose gel electrophoresis to reveal a short isoform of a *DUOX2* transcript (DUOX2b) carrying a G allele in mutant pigs. The RT-PCR amplicon lengths of *DUOX2a* and *DUOX2b* were 377 bp and 274 bp, respectively. (C) Identification of the *DUOX2b* isoform using Sanger sequencing of *DUOX2* cDNA amplicons. (D) The WT *DUOX2* gene produces a WT full-length protein composed of 1535 amino acids (DUOX2 WT). A mutant full-length protein (DUOX2a), as well as a truncated protein (DUOX2b), was detected in mutant pigs. The c.1226 A>G mutation, with its flanking sequences, was predicted as a potential ESE motif using ESEfinder software, and the motif sequence carrying the mutant G allele presented a higher weighted majority vote (WMV) score than that carrying the A allele (3.19 versus 2.82), suggesting that the G allele potentially increased binding affinity for SRSF2 (a member of the family of pre-mRNA splicing factors) and contributed to a high incidence of alternative splicing (AS). Moreover, the AS, resulting in exon 10 skipping, introduces a premature stop codon and leads to the generation of a truncated protein (DUOX2b). The protein structures were generated via SWISS-MODEL server (https://swissmodel.expasy.org/). Green boxes, ESE motif; red stars, D409G mutation. (E) The structure of the minigene containing exon 9, intron9, exon 10 (the ESE motif included), intron 10 and exon 11 of *DUOX2*. (F) cDNA from transfected minigenes was amplified by plasmid-specific primers, and RT-PCR products were analyzed by 2% agarose gel electrophoresis. The results revealed that exon 10 (103 bp) in the mutant minigene was skipped completely and produced a shorter transcript (424 bp) compared with the WT minigene (527 bp or 424 bp), which had only partial skipping of exon 10. Lane 1, marker; Lane 2, WT minigene with the 527 bp upper band (91 bp+103 bp+164 bp+169 bp) and 424 bp lower band (91 bp+164 bp+169 bp); Lane 3, mutant minigene (G allele) with a 424 bp band (91 bp+164 bp+169 bp); Lane 4, empty 169 bp plasmid. CMV, cytomegalovirus.

Because the p.D409G mutation was located in the peroxidase-like domain of DUOX2, which is essential for the hydrogen peroxide (H_2_O_2_)-generating activity of DUOX2 (Grasberger et al., 2007), we hypothesized that the p.D409G mutation might impair H_2_O_2_ production directly. To test the hypothesis, HeLa cells were co-transfected with either WT or D409G DUOX2 vectors in the presence of DUOXA2 (the activator protein of DUOX2). The results showed that the H_2_O_2_ concentration was significantly reduced in the D409G *DUOX2* group (Fig. 4A), indicating the p.D409G mutation does affect H_2_O_2_ generation. Collectively, these results suggest that the p.D409G substitution impairs H_2_O_2_ production and is responsible for the thyroid hormone deficiency in pigs.

### The *DUOX2* c.1226 A>G mutation is located within an ESE and causes aberrant splicing of *DUOX2* transcripts

To determine the consequences of the *DUOX2* c.1226 A>G mutation at the transcript level, a 377 bp sequence flanking the mutation site was amplified by reverse transcription PCR (RT-PCR) in samples collected from WT and mutant thyroids. Results showed that pigs with genotypes of AG and GG had an additional 274 bp transcript that we dubbed *DUOX2b* (Fig. 4B). This finding suggests that the mutant G allele might cause aberrant splicing. Sanger sequencing further confirmed that the entire exon 10 (103 bp) was skipped during splicing to generate the *DUOX2b* transcript (Fig. 4C), which resulted in the truncated amino acid sequence due to a frameshift and a premature stop codon (Fig. 4C; Fig. S1). To test whether the *DUOX2* c.1226 A>G mutation affected the transcript splicing, the ESEfinder server was used to predict potential ESE elements in the *DUOX2* gene, because previous reports have suggested that mutations in ESE could cause exon skipping or aberrant splicing (Sun et al., 1993)*.* Interestingly, the c.1226 A>G mutation site with its flanking sequences (G [A/G] TCTGAGG) was predicted as a potential ESE motif, and a higher weighted majority vote (WMV) score of 3.19 was observed when the motif sequence carried the mutant G allele. These data indicate that the mutant ESE motif might potentially increase the affinity for SRSF2 (a member of the family of pre-mRNA splicing factors) and promote skipping of exon 10 (Fig. 4D). To investigate this possibility, we constructed a minigene containing the genomic sequence of exon 9 to exon 11 of *DUOX2*, including the ESE motif, and transfected WT or mutant (A/G transition) minigenes into HeLa cells (Fig. 4E). After 24 h, RT-PCR fragments of the minigenes were amplified and were subjected to Sanger sequencing. The *in vitro* results revealed that exon 10 (103 bp) in the mutant minigene was skipped completely and produced the shorter transcript *DUOX2b* (424 bp), compared with the WT minigene (527 bp or 424 bp), with partial skipping of exon 10, indicating that the alternative splicing is slightly enhanced with an A allele and greatly enhanced with a mutant G allele (Fig. 4F). To assess whether the truncated DUOX2b protein was also associated with H_2_O_2_ generation, WT full-length *DUOX2*, mutant full-length *DUOX2a* and the truncated *DUOX2b* transcripts were transfected into HeLa cells to characterize H_2_O_2_ production. To assess whether *DUOX2b* was also associated with H_2_O_2_ generation, WT or mutant full-length *DUOX2b* transcripts were transfected into HeLa cells to characterize H_2_O_2_ production. The results showed that the truncated mutant *DUOX2b* transcript significantly decreased H_2_O_2_ production compared with the WT full-length *DUOX2* transcript (Fig. 4A). Moreover, due to the loss of more functional domains, DUOX2b produced significantly less H_2_O_2_ than DUOX2a (Fig. 4A). Taken together, we conclude that the A/G transition enhanced the activity of the ESE in *DUOX2* and thus contributed to complete exon 10 skipping, which might lead to severe thyroid hormone deficiency through decreased H_2_O_2_ production.

## DISCUSSION

This study demonstrates that whole-exome sequencing integrated with family-based GWAS is a robust approach to reveal the causative mutation in ENU-induced mutant pigs. Moreover, we identified that the causal *DUOX2* mutation, p.D409G, located within an ESE domain, could give rise to aberrant splicing of *DUOX2* transcripts, which might lead to severe thyroid hormone deficiency due to the lower level of H_2_O_2_ production in pigs. These results suggest the D409G mutant pigs could potentially be used as a large animal model for human thyroid diseases.

ENU mutagenesis, a phenotype-driven approach, has been used for more than two decades in mice and zebrafish to study gene function or mimic human diseases; however, ENU mutagenesis in large animals such as pigs has never been reported. The urgent need to create large animal models for human disease inspired us to perform ENU mutagenesis screens in pigs (Hai et al., 2017). Overall, ENU mutagenesis has many advantages because no *priori* assumptions concerning the gene functions have to be made, and ENU mutagenesis can induce a series of point mutations at the whole-genome level, which more accurately mimics the molecular spectrum than a gene deletion model of human disease (Oliver and Davies, 2012). In our mutagenesis project, a wealth of mutants was produced, which might provide a rich mutant resource to exploit the full potential of this model organism. Identification of the causative mutations of these mutants could pave the way for dissecting gene function or uncovering novel pathways and genes, and thus would substantially extend our knowledge of the biological basis underlying the phenotypes.

One of the major rate-limiting steps in ENU mutagenesis studies is the identification of causative mutations in ENU-mutagenized pedigrees, with challenges most likely due to the heterogeneous genetic background of ENU-induced mutants, the relatively smaller sample size of mutant pedigrees and the low resolution of genetic mapping. With regard to gene mapping analysis such as GWAS and linkage analysis, the process crucially depends on the extent and pattern of linkage disequilibrium (LD) in the genome (Carlson et al., 2004), which is estimated by genome-wide SNP panels. In 2009, a high-density porcine SNP chip (60K), which was designed mainly on SNP data from Western pig breeds (Ramos et al., 2009), became commercially available and has been widely used to identify genes for qualitative and quantitative traits (Sharma et al., 2015; Goddard and Hayes, 2009). However, a previous study confirmed the divergent evolution between Chinese native and Western pigs, and revealed that the LD extent was much longer in Western pigs than that in Chinese pigs, implying that higher marker density would be required to capture LD in causal variants with GWAS on Chinese pigs (Ai et al., 2013). These findings suggest that low SNP density and sparse marker coverage would result in low resolution for LD measure, thus significantly reducing the gene mapping efficiency. In this study, family-based GWAS revealed a unique significant association signal extending over 40 Mb and around 374 genes located within this region, suggesting that isolating the causative mutation from these candidate genes using traditional candidate gene methods would be a challenge. As an alternative method, we chose to determine whether whole-exome sequencing, which is more cost-effective than whole-genome sequencing, was a highly efficient approach to identify the causal mutations in pigs. In our analysis, we used a recently designed exome capture kit, based on the Ensembl annotation of assembly version 10.2 of the pig genome (Robert et al., 2014). As expected, our findings demonstrated that, combined with GWAS, whole-exome sequencing is an efficient and rapid method for variant detection. We were able to quickly narrow down the 374 candidate genes to only seven candidate mutations in our mutant pedigree.

As previously mentioned, a missense mutation in the *DUOX2* gene (p.D409G) was identified to completely co-segregate with the mutant trait. In humans, *DUOX2* gene mutations can produce a spectrum of congenital hypothyroidism with an autosomal recessive inheritance (Targovnik et al., 2016; Ohye and Sugawara, 2010). Here, the mutant pigs, presenting low T4 levels and high TSH levels, resembled the phenotype observed in human patients carrying the *DUOX2* gene mutations. In addition, consistent with findings in human patients with congenital hypothyroidism, our results indicated that the *DUOX2* gene variant can lead to a severe disruption of H_2_O_2_-generating activity (Kizys et al., 2017). Thus, this mutant pedigree would be a potential animal model for human thyroid dysfunction, and, in particular, the nude skin phenotype might extend our knowledge about hair loss in human patients with thyroid-related problems (Vincent and Yogiraj, 2013). Typically, point mutations in the exonic regions of genes are traditionally assumed to exert their effects by altering amino acids in the encoded proteins (Cartegni et al., 2002). However, increasing evidence shows that many human genetic diseases are caused by exonic mutations, which are relevant to pre-mRNA splicing and are always identified as ESE mutations (Blencowe, 2000; Cáceres and Kornblihtt, 2002). Previous studies have also demonstrated that ESE are specifically recognized by one or more SR proteins, which are a family of highly conserved serine/arginine-rich RNA-binding proteins that are implicated in the assembly of splicing complexes (Sun et al., 1993; Tian and Maniatis, 1993; Gontarek and Derse, 1996; Liu et al., 1998). Notably, in the present study, the mutant G allele presented a higher WMV score using *in silico* analysis, indicating that the mutation and flanking sequences might be within a potential ESE motif. Consistent with the bioinformatics prediction, our *in vitro* analysis demonstrated that the G allele could produce alternative splicing of *DUOX2* transcripts. However, further work will be required to reveal the roles of the truncated *DUOX2* transcript in the pathogenesis of thyroid dysfunction, which is essential for modeling thyroid disease in humans.

In summary, we have demonstrated that exome sequencing integrated with family-based GWAS is a cost-effective approach to identify causal mutation in ENU-induced mutant pedigrees. Our data indicate that an ESE mutation in the *DUOX2* gene (p.D409G) was responsible for the severe phenotype of our mutants. In light of our findings, we suggest that the mutant caused by the D409G mutation in *DUOX2* would provide a valuable resource for the study of molecular pathogenesis and possible treatments for human congenital hypothyroidism.

## MATERIALS AND METHODS

### Animals

The Bama pigs used in this study were fed *ad libitum* with a commercial pig diet (nutrient levels according to the United States National Research Council) and water throughout the experimental period. All experiments involving animals were performed according to the protocols approved by the Institutional Animal Care and Use Committee of the Institute of Zoology, Chinese Academy of Sciences, China.

### Genotyping, quality control and family-based whole-genome association analysis

Genomic DNA was isolated from ear tissues using a routine phenol/chloroform extraction, and whole-genome SNP genotyping was performed using porcine SNP60 BeadChips (Illumina, San Diego, CA, USA) containing 62,163 SNP markers at Beijing Compass Biotechnology (Beijing, China). SNP genotyping data were processed by removing SNPs with a call rate <90% for all animals, with minor allele frequencies (MAF) <0.05, and with a *P*-value of χ^2^ test for a Hardy–Weinberg equilibrium <1.0×10^−3^. After applying these quality control measures, 22,610 SNPs were retained for subsequent association analyses. Family-based whole-genome association studies were performed using the transmission disequilibrium test implemented with the PLINK tool (http://zzz.bwh.harvard.edu/plink/index.shtml). Multiple-testing corrected *P*-values can be obtained from the permutation tests.

### Exome capture and massively parallel sequencing

The genomic DNA from ear tissues was fragmented by Covaris technology, sequencing libraries containing fragments of 200-300 bp were constructed, and adapters were ligated to both ends of the fragments for each library. Then, the libraries were subjected to pre-capture PCR, hybridization and capture using the Roche Nimblegen SeqCAP EZ system, which targets ∼60.6 Mb of coding regions. Sequencing was performed on the Illumina HiSeq 2500 platform, generating 126 bp paired-end reads.

### Sequence alignment, variant calling and filtering

The raw sequence reads were split based on index and the adapters were trimmed out. A trimmomatic program was first used to remove adapter contamination and trim sequencing reads with low-quality bases. The remaining qualified reads were then mapped to pig build 10.2 reference sequence using Burrows–Wheeler Aligner (BWA) tools (http://bio-bwa.sourceforge.net/) with default parameters. The sequence alignment map (SAM) files (generated from BWA) that contained the read alignments were converted into binary alignment map (BAM) files, and the processed BAM files (sorting and removing duplicates) were then used to call variants with the SAMtools program (http://samtools.sourceforge.net/). The BEDTools software package (https://bedtools.readthedocs.io/en/latest/) was used for analyzing the coverage distributions. Mutation filtering was performed according to a designed procedure described in Fig. 1D.

### Sanger resequencing

To investigate whether the mutations detected by exome sequencing were co-segregated with the mutant phenotype, all identified variants were confirmed by PCR amplification and DNA Sanger sequencing for all members in the whole pedigree. PCR primers were designed using the Primer3 server (http://frodo.wi.mit.edu/). The PCR products were verified by 1.5% agarose gel electrophoresis. Sanger sequencing was performed using an ABI 3100/3130 DNA analyzer.

### Histopathology

Tissues were fixed with 4% neutral buffered formalin, and then embedded in paraffin according to standard laboratory procedures. Hematoxylin-Eosin staining was performed on 5-μm-thick sections of paraffin-embedded tissues.

### Thyroid hormone measurement

Blood samples were obtained by puncture of the pre-caval vein. Serum from clot-activator-treated blood was separated at 751 ***g*** for 5 min in a refrigerated centrifuge set at 4°C. Serum total T4, T3 and TSH concentrations were measured by chemiluminescent immunoassay using commercial kits (Siemens Healthcare Diagnostics, Munich, Germany) and the ADVIA Centaur XP Immunoassay System (Siemens Healthcare Diagnostics) following the manufacturer's instructions.

### RNA isolation and RT-PCR

Total RNA from tissues or cells was isolated and purified using Trizol (Thermo Fisher Scientific, Carlsbad, CA, USA) and then reversely transcribed to complementary DNA (cDNA) using a FastQuant RT kit (Tiangen Bio, Beijing, China) according to the manufacturer's instructions. We amplified cDNA from thyroids or the HeLa-cell-transfected minigenes with 2×Taq PCR MasterMix (Tiangen Bio) according to the protocols of the manufacturer, and the PCR products were analyzed by gel electrophoresis on a 2% agarose gel. pDNA3.1-specific primers were used for minigenes. All primer sequences are shown in Table S4.

### Vector construction

The RNA from thyroid was reversely transcribed to cDNA using M-MLV reverse transcriptase (Promega, Madison, WI, USA). We amplified the open reading frames (ORFs) of *DUOX2* and *DUOXA2* genes using Phusion High-Fidelity PCR Master Mix (New England BioLabs, Beverly, MA, USA). The inserts were directionally cloned into the *Eco*RI and *Sac*II restriction sites of the pcDNA3.1 vector. *DUOX2* minigenes were created by Phusion High-Fidelity PCR amplification of genomic DNA. PCR products were digested with *Hind*III and *Eco*RI and then cloned into *Hind*III and *Eco*RI sites in the pcDNA3.1 vector. All vectors constructed were confirmed by Sanger sequencing. All primer sequences are shown in Table S4.

### Cell culture and transfection

HeLa cells were cultured in Dulbecco's modified Eagle medium with 10% fetal bovine serum in a humidified 5% CO_2_ incubator at 37°C, and then seeded in six-well plates at a concentration of ∼300,000 per well. Then, the cells were incubated for 24 h to reach cell densities of 70% confluence. For H_2_O_2_ assays, HeLa cells were transfected with 2 μg WT DUOX2 or D409G DUOX2 in the presence or absence of 500 ng DUOXA2 using Fugene HD transfection reagent (Promega). GFP expression from a co-transfected pEGFP vector (1:5 relative to the DUOX2 and DUOXA2 plasmids) was used to monitor transfection efficiency. For each minigene experiment, 3 μg plasmid was transfected, and cells were collected for RNA isolation after 24 h.

### H_2_O_2_ concentration

HeLa cells grown in six-well plates were tested for H_2_O_2_ generation using an Amplex Red H_2_O_2_ assay kit (Thermo Fisher Scientific). At 48 h after transfection, cell monolayers were incubated with 0.1 U/ml horseradish peroxidase and 50 M Amplex Red reagent. Then, 1 h after incubation at 37°C, the medium was collected and fluorescence intensity was measured, with excitation wavelength at 535 nm and emission wavelength at 595 nm, on a Synergy 4 Multi-Mode Microplate Reader (BioTek Instruments, Winooski, VT, USA). H_2_O_2_ concentrations were calculated based on changes in fluorescence intensity converted into absolute micromoles or nanomoles of H_2_O_2_ using a calibration curve. The calibration curve was obtained by known concentrations of H_2_O_2_ run in the same experiment, and all fluorescence measurements were corrected for autofluorescence of the medium. The H_2_O_2_ levels were normalized to the total protein content in the corresponding samples.

### Statistics

Student's *t*-tests (two-tailed) were applied to determine the statistical significance of differences between groups using GraphPad Prism 5. *P*-values <0.05 were considered significant. The applied statistical method and replicates can be found in the figure legends.

## Supplementary Material

Supplementary information

## References

[DMM036616C1] Acevedo-Arozena A., Wells S., Potter P., Kelly M., Cox R. D. and Brown S. D. M. (2008). ENU mutagenesis, a way forward to understand gene function. *Annu. Rev. Genomics Hum. Genet.* 9, 49-69. 10.1146/annurev.genom.9.081307.164224 18949851

[DMM036616C2] Ai H., Huang L. and Ren J. (2013). Genetic diversity, linkage disequilibrium and selection signatures in chinese and Western pigs revealed by genome-wide SNP markers. *PLoS ONE* 8, e56001 10.1371/journal.pone.0056001 23409110 PMC3567019

[DMM036616C3] Blencowe B. J. (2000). Exonic splicing enhancers: mechanism of action, diversity and role in human genetic diseases. *Trends Biochem. Sci.* 25, 106-110. 10.1016/S0968-0004(00)01549-8 10694877

[DMM036616C4] Boycott K. M., Vanstone M. R., Bulman D. E. and Mackenzie A. E. (2013). Rare-disease genetics in the era of next-generation sequencing: discovery to translation. *Nat. Rev. Genet.* 14, 681-691. 10.1038/nrg3555 23999272

[DMM036616C5] Cáceres J. F. and Kornblihtt A. R. (2002). Alternative splicing: multiple control mechanisms and involvement in human disease. *Trends Genet.* 18, 186-193. 10.1016/S0168-9525(01)02626-9 11932019

[DMM036616C6] Carlson C. S., Eberle M. A., Rieder M. J., Yi Q., Kruglyak L. and Nickerson D. A. (2004). Selecting a maximally informative set of single-nucleotide polymorphisms for association analyses using linkage disequilibrium. *Am. J. Hum. Genet.* 74, 106-120. 10.1086/381000 14681826 PMC1181897

[DMM036616C7] Cartegni L., Chew S. L. and Krainer A. R. (2002). Listening to silence and understanding nonsense: exonic mutations that affect splicing. *Nat. Rev. Genet.* 3, 285-298. 10.1038/nrg775 11967553

[DMM036616C8] Choi C. M., Vilain S., Langen M., Van Kelst S., De Geest N., Yan J., Verstreken P. and Hassan B. A. (2009). Conditional mutagenesis in Drosophila. *Science* 324, 54 10.1126/science.1168275 19342580

[DMM036616C9] De Stasio E. A. and Dorman S. (2001). Optimization of ENU mutagenesis of Caenorhabditis elegans. *Mutat. Res.* 495, 81-88. 10.1016/S1383-5718(01)00198-X 11448645

[DMM036616C10] Fairfield H., Gilbert G. J., Barter M., Corrigan R. R., Curtain M., Ding Y., D'ascenzo M., Gerhardt D. J., He C., Huang W., et al. (2011). Mutation discovery in mice by whole exome sequencing. *Genome Biol.* 12, R86 10.1186/gb-2011-12-9-r86 21917142 PMC3308049

[DMM036616C11] Goddard M. E. and Hayes B. J. (2009). Mapping genes for complex traits in domestic animals and their use in breeding programmes. *Nat. Rev. Genet.* 10, 381-391. 10.1038/nrg2575 19448663

[DMM036616C12] Goh G. and Choi M. (2012). Application of whole exome sequencing to identify disease-causing variants in inherited human diseases. *Genomics Inform.* 10, 214-219. 10.5808/GI.2012.10.4.214 23346032 PMC3543920

[DMM036616C13] Gontarek R. R. and Derse D. (1996). Interactions among SR proteins, an exonic splicing enhancer, and a lentivirus Rev protein regulate alternative splicing. *Mol. Cell. Biol.* 16, 2325-2331. 10.1128/MCB.16.5.2325 8628299 PMC231220

[DMM036616C14] Grasberger H., De Deken X., Miot F., Pohlenz J. and Refetoff S. (2007). Missense mutations of dual oxidase 2 (DUOX2) implicated in congenital hypothyroidism have impaired trafficking in cells reconstituted with DUOX2 maturation factor. *Mol. Endocrinol.* 21, 1408-1421. 10.1210/me.2007-0018 17374849

[DMM036616C15] Hai T., Cao C., Shang H., Guo W., Mu Y., Yang S., Zhang Y., Zheng Q., Zhang T., Wang X. et al. (2017). Pilot study of large-scale production of mutant pigs by ENU mutagenesis. *eLife* 6, e26248 10.7554/eLife.26248 28639938 PMC5505698

[DMM036616C16] Hrabe De Angelis M. H., Flaswinkel H., Fuchs H., Rathkolb B., Soewarto D., Marschall S., Heffner S., Pargent W., Wuensch K., Jung M. et al. (2000). Genome-wide, large-scale production of mutant mice by ENU mutagenesis. *Nat. Genet.* 25, 444-447. 10.1038/78146 10932192

[DMM036616C17] Jamuar S. S. and Tan E.-C. (2015). Clinical application of next-generation sequencing for Mendelian diseases. *Hum. Genomics* 9, 10 10.1186/s40246-015-0031-5 26076878 PMC4482154

[DMM036616C18] Kizys M. M. L., Louzada R. A., Mitne-Neto M., Jara J. R., Furuzawa G. K., de Carvalho D. P., Dias-da-Silva M. R., Nesi-França S., Dupuy C. and Maciel R. M. B. (2017). DUOX2 mutations are associated with congenital hypothyroidism with ectopic thyroid gland. *J. Clin. Endocrinol. Metab.* 102, 4060-4071. 10.1210/jc.2017-00832 28666341

[DMM036616C19] Lai L., Kolber-Simonds D., Park K. W., Cheong H. T., Greenstein J. L., Im G. S., Samuel M., Bonk A., Rieke A., Day B. N., et al. (2002). Production of alpha-1,3-galactosyltransferase knockout pigs by nuclear transfer cloning. *Science* 295, 1089-1092. 10.1126/science.1068228 11778012

[DMM036616C20] Liu H.-X., Zhang M. and Krainer A. R. (1998). Identification of functional exonic splicing enhancer motifs recognized by individual SR proteins. *Genes Dev.* 12, 1998-2012. 10.1101/gad.12.13.1998 9649504 PMC316967

[DMM036616C21] Moreno J. C., Bikker H., Kempers M. J. E., Van Trotsenburg A. S. P., Baas F., De Vijlder J. J. M., Vulsma T. and Ris-Stalpers C. (2002). Inactivating mutations in the gene for thyroid oxidase 2 (THOX2) and congenital hypothyroidism. *N. Engl. J. Med.* 347, 95-102. 10.1056/NEJMoa012752 12110737

[DMM036616C22] Nunoya T., Shibuya K., Saitoh T., Yazawa H., Nakamura K., Baba Y. and Hirai T. (2007). Use of miniature pig for biomedical research, with reference to toxicologic studies. *J. Toxicol. Pathol.* 20, 125-132. 10.1293/tox.20.125

[DMM036616C23] Ohye H. and Sugawara M. (2010). Dual oxidase, hydrogen peroxide and thyroid diseases. *Exp. Biol. Med. (Maywood)* 235, 424-433. 10.1258/ebm.2009.009241 20407074

[DMM036616C24] Oliver P. L. and Davies K. E. (2012). New insights into behaviour using mouse ENU mutagenesis. *Hum. Mol. Genet.* 21, R72-R81. 10.1093/hmg/dds318 22892373 PMC3459650

[DMM036616C25] Prather R. S., Lorson M., Ross J. W., Whyte J. J. and Walters E. (2013). Genetically engineered pig models for human diseases. *Annu. Rev. Anim. Biosci.* 1, 203-219. 10.1146/annurev-animal-031412-103715 25387017 PMC4460601

[DMM036616C26] Ramos A. M., Crooijmans R. P. M. A., Affara N. A., Amaral A. J., Archibald A. L., Beever J. E., Bendixen C., Churcher C., Clark R., Dehais P., et al. (2009). Design of a high density SNP genotyping assay in the pig using SNPs identified and characterized by next generation sequencing technology. *PLoS ONE* 4, e6524 10.1371/journal.pone.0006524 19654876 PMC2716536

[DMM036616C27] Robert C., Fuentes-Utrilla P., Troup K., Loecherbach J., Turner F., Talbot R., Archibald A. L., Mileham A., Deeb N., Hume D. A. et al. (2014). Design and development of exome capture sequencing for the domestic pig (Sus scrofa). *BMC Genomics* 15, 550 10.1186/1471-2164-15-550 24988888 PMC4099480

[DMM036616C28] Schneeberger K. (2014). Using next-generation sequencing to isolate mutant genes from forward genetic screens. *Nat. Rev. Genet.* 15, 662-676. 10.1038/nrg3745 25139187

[DMM036616C29] Sharma A., Lee J. S., Dang C. G., Sudrajad P., Kim H. C., Yeon S. H., Kang H. S. and Lee S.-H. (2015). Stories and challenges of genome wide association studies in livestock - a review. *Asian-Australas. J. Anim. Sci.* 28, 1371-1379. 10.5713/ajas.14.0715 26194229 PMC4554843

[DMM036616C30] Sun Q., Mayeda A., Hampson R. K., Krainer A. R. and Rottman F. M. (1993). General splicing factor SF2/ASF promotes alternative splicing by binding to an exonic splicing enhancer. *Genes Dev.* 7, 2598-2608. 10.1101/gad.7.12b.2598 8276242

[DMM036616C31] Swindle M. M., Makin A., Herron A. J., Clubb F. J. Jr. and Frazier K. S. (2012). Swine as models in biomedical research and toxicology testing. *Vet. Pathol.* 49, 344-356. 10.1177/0300985811402846 21441112

[DMM036616C32] Targovnik H. M., Citterio C. E., Siffo S. and Rivolta C. M. (2016). Advances and perspectives in genetics of congenital thyroid disorders. *J. Clin. Mol. Endocrinol.* 1, 23 http://clinical-and-molecular-endocrinology.imedpub.com/advances-and-perspectives-in-genetics-of-congenital-thyroid-disorders.pdf

[DMM036616C33] Tian M. and Maniatis T. (1993). A splicing enhancer complex controls alternative splicing of doublesex pre-mRNA. *Cell* 74, 105-114. 10.1016/0092-8674(93)90298-5 8334698

[DMM036616C34] Vincent M. and Yogiraj K. (2013). A descriptive study of alopecia patterns and their relation to thyroid dysfunction. *Int. J. Trichology* 5, 57-60. 10.4103/0974-7753.114701 23960405 PMC3746235

[DMM036616C35] Wienholds E., Van Eeden F., Kosters M., Mudde J., Plasterk R. H. and Cuppen E. (2003). Efficient target-selected mutagenesis in zebrafish. *Genome Res.* 13, 2700-2707. 10.1101/gr.1725103 14613981 PMC403812

[DMM036616C36] Zhao J., Xu W., Ross J. W., Walters E. M., Butler S. P., Whyte J. J., Kelso L., Fatemi M., Vanderslice N. C., Giroux K., et al. (2015). Engineering protein processing of the mammary gland to produce abundant hemophilia B therapy in milk. *Sci. Rep.* 5, 14176 10.1038/srep14176 26387706 PMC4585688

