# Supplementary material for: An exonic splicing enhancer mutation in DUOX2 causes aberrant alternative splicing and severe congenital hypothyroidism in Bama pigs
Source: Dis Model Mech. 2019 Jan 15;12(1):dmm036616. doi: 10.1242/dmm.036616 (PMC6361156; doi:10.1242/dmm.036616)
Supplement: Supplementary information [file dmm-12-036616-s1.pdf]

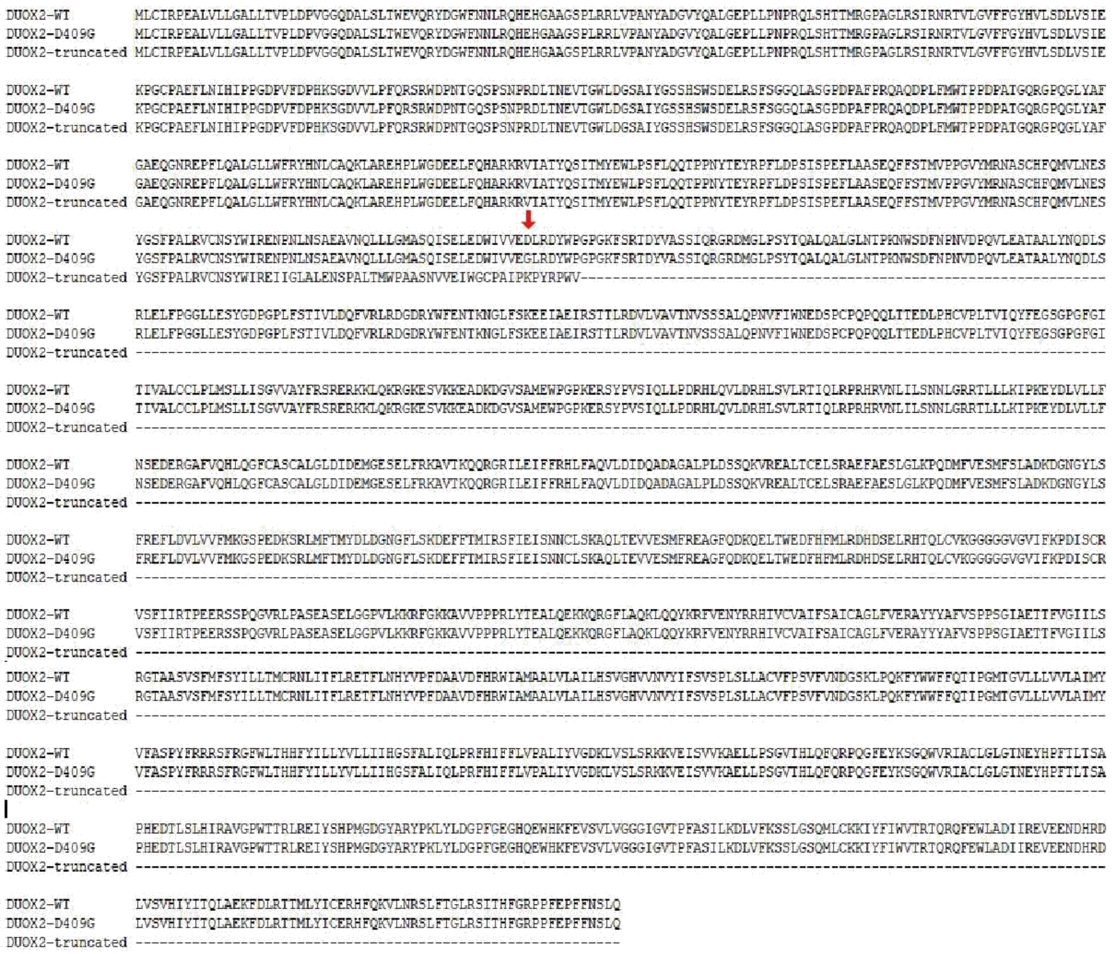

Figure S1. Sequence alignment of DUOX2-WT, DUOX2-D409G, and DUOX2-truncated proteins. The p. D409G mutation site was marked by red arrow. The exon 10 skipping causes frame shift and introduces a premature stop codon, leading to generate a truncated protein.

Disease Models & Mechanisms • Supplementary information

Table S1. The pigs in the mutant pedigree were selected for genotyping using 60K chips

Family ID	Pig ID	Gender	Father (boar)	Mother (sow)	Phenotype
I	005T118	♂	W000a199	TBB005	WT
	1220106	♀	005T118	023T063	WT
	1327403	♀	005T118	1220106	WT
	1327404	♂	005T118	1220106	WT
	1327407	♀	005T118	1220106	Mut
	1327408	♀	005T118	1220106	Mut
	1452001	♂	005T118	1220106	Mut
	1452002	♀	005T118	1220106	Mut
	1452003	♂	005T118	1220106	Mut
	1452005	♂	005T118	1220106	WT
	1452006	♂	005T118	1220106	Mut
	1452007	♂	005T118	1220106	WT
	1452008	♀	005T118	1220106	WT
	1452010	♂	005T118	1220106	WT
II	1220103	♀	005T118	023T063	WT
	1327601	♀	005T118	1220103	WT
	1327603	♀	005T118	1220103	WT
	1327607	♂	005T118	1220103	Mut
	1453401	♂	005T118	1220103	WT
	1453404	♂	005T118	1220103	WT
	1453406	♀	005T118	1220103	WT
	1453407	♂	005T118	1220103	Mut
	1453408	♀	005T118	1220103	Mut
III	1220104	♀	005T118	023T063	WT
	1345501	♀	005T118	1220104	WT
	1345503	♀	005T118	1220104	WT
	1345504	♀	005T118	1220104	Mut
	1345505	♂	005T118	1220104	Mut
IV	1207702	♀	005T118	ZBBW11000126	WT
	1341602	♂	005T118	1207702	WT
	1341603	♂	005T118	1207702	WT
	1341604	♂	005T118	1207702	WT
	1341605	♂	005T118	1207702	Mut
	1341606	♀	005T118	1207702	Mut
	1451301	♂	005T118	1207702	Mut
	1451302	♀	005T118	1207702	Mut
	1451304	♂	005T118	1207702	WT
	1451305	♀	005T118	1207702	WT
	1451307	♂	005T118	1207702	WT

Disease Models & Mechanisms • Supplementary information

Table S2. Family-based genome wide association studies identified loci showing significant association (*P*_permutation<0.01)

Click here to Download Table S2

Table S3. The frequency and distribution of *DUOX2* c.1226 A>G mutation within the mutant pedigree and other pig breeds

Group	Phenotype	number	AA	AG	GG	A%	G%
Mutant pedigree	Mutant	25	0	0	25	55.3%	44.7%
	WT	97	38	59	0		
Bama	WT	128	128	0	0	100%	0
Large White	WT	55	55	0	0	100%	0
Landrace	WT	50	50	0	0	100%	0

Table S4. Primer sequences used in the present study

Primers	Sequences	Assays
8-1F	ATTACACAGAGTACCGGCCTT	RT-PCR
8-1R	CCCCATATCTCGACCACGTT	RT-PCR
pCDNA3.1-F	CTAGAGAACCCACTGCTTAC	RT-PCR
BGH-R	TAGAAGGCACAGTCGAGG	RT-PCR
DUOX2-3F	TTTGAATTCATGCTCTGCATAAGACCAGAGGCAC	H2O2 assay
DUOX2-3R	TTTCCGCGGAAAGTTCTCATAGTGGTGCACAAAGTGGG	H2O2 assay
DUOXA2-1F	TTCGAATTCATGACTCTGTGGAACGGCGTG	H2O2 assay
DUOXA2-1R	TTTCCGCGGCAGGTTAGTACTGATGGTTAAG	H2O2 assay
Minigene-DUOX2-1F	TTTAAGCTTAAATGCTAGCTGTCATTTCCAGATGGTC	minigene
Minigene-DUOX2-1R	TTTGAATTCCTGAGGGTCCACGTTGGGATTGAA	minigene

Disease Models & Mechanisms • Supplementary information
